# Immune reconstitution in AML and MDS patients undergoing allogeneic hematopoietic cell transplantation (allo-HCT) with treosulfan- or TBI-based conditioning

**DOI:** 10.1038/s41409-025-02702-2

**Published:** 2025-08-22

**Authors:** Lina Kolloch, Philipp Berning, Jörn C. Albring, Julian Ronnacker, Christian Reicherts, Simon Call, Julia Marx, Matthias Floeth, Eva Esseling, Hans Theodor Eich, Christoph Schliemann, Jan-Henrik Mikesch, Georg Lenz, Matthias Stelljes

**Affiliations:** 1 Department of Hematology and Oncology, University Hospital Muenster, Muenster, Germany; 2 Department of Radiation Oncology, University Hospital Muenster, Muenster, Germany

**Keywords:** Acute myeloid leukaemia, Myelodysplastic syndrome, Stem-cell therapies, Chemotherapy, Radiotherapy

## To the Editor:

Cellular and humoral immune reconstitution after allo-HCT impacts infections, graft-versus-host disease (GvHD) and relapse-risk. How conditioning regimens affect immune recovery is largely unknown. A study comparing fludarabine/treosulfan (FluTreo) and fludarabine/total body irradiation (FluTBI) conditioning regimens showed no significant differences in overall survival (OS) and relapse-free survival (RFS) or GvHD incidence [1]. However, the non-relapse mortality (NRM) rate was lower among patients receiving FluTreo. An EBMT-data-based study also demonstrated higher NRM in older patients treated with FluTBI, whereas relapse rates were lower for that regimen [2].

We retrospectively analyzed the immune reconstitution of 311 patients with acute myeloid leukemia (AML) or myelodysplastic syndrome (MDS) who underwent allo-HCT following FluTreo or FluTBI conditioning (Supplementary Table 1).

## Cellular immune reconstitution

Comprehensive data on cellular immune reconstitution (CD4+ and CD8+ T cell and CD19+ cell counts) post-transplant were available for 279 patients (90%), with baseline characteristics presented in Supplementary Table 2, and examined at multiple time points.

At one-year post-transplant, 90% of patients exhibited normalized CD3+/CD8+ and CD19+ lymphocyte, whereas CD3+/CD4+ T cell counts increased but did not fully reconstitute. Notably, CD3+/CD4+ normalization differed significantly between FluTreo and FluTBI conditioning groups, with a higher proportion of FluTreo patients achieving normalization by day +180 (Supplementary Fig. 1a–c, e–g).

Multivariate regression analysis of lymphocyte normalization by day +180 (Table 1) revealed that CD3+/CD4+ reconstitution was significantly associated with FluTreo and donor cytomegalovirus (CMV) seropositivity, but negatively correlated to the use of in-vivo T-cell depletion (TCD). CD3+/CD8+ normalization was associated with donor CMV seropositivity, and ECOG performance score (PS) <2. CD19+ normalization was linked to unrelated donors and negatively correlated to complex karyotypes.

**Table 1:** Multivariate analysis for normalization of lymphocyte subpopulations by day +180/IgG levels at two years after allo-HCT.

	CD3+/CD4+ lymphocytes normalized by day +180		CD3+/CD8+ lymphocytes normalized by day +180		CD19+ lymphocytes normalized by day +180		IgG normalized at two years	
	HR (95% CI for HR)	*p*-value	HR (95% CI for HR)	*p*-value	HR (95% CI for HR)	*p*-value	HR (95% CI for HR)	*P*-value
Sex female vs. male	0.83 (0.44–1.57)	0.565	0.92 (0.67–1.27)	0.624	1.21 (0.83–1.76)	0.329	1.13 (0.85–1.50)	0.415
FluTBI vs. FluTreo	3.42 (1.12–10.49)	0.031	1.02 (0.65–1.60)	0.944	0.81 (0.48–1.39)	0.448	1.54 (1.03–2.30)	0.034
Age > 60 years before allo-HCT	0.51 (0.25–1.05)	0.069	0.88 (0.60–1.28)	0.498	1.14 (0.72–1.81)	0.581	1.01 (0.72–1.42)	0.938
Diagnosis AML vs. MDS	0.68 (0.28–1.69)	0.409	1.12 (0.75–1.69)	0.576	1.10 (0.69–1.74)	0.693	1.18 (0.87–1.61)	0.280
ECOG PS (0-1 vs. >1)	0.44 (0.14–1.34)	0.150	0.54 (0.32–0.93)	0.025	1.15 (0.61–2.15)	0.672	1.19 (0.85–1.65)	0.314
HCT-CI score grouped (0–2, 3–5, >5)	1.47 (0.80–2.71)	0.214	1.03 (0.79–1.34)	0.819	0.89 (0.65–1.21)	0.457	0.74 (0.58–0.93)	0.011
Patient CMV− (vs. CMV+)	2.05 (0.93–4.53)	0.076	1.12 (0.80–1.59)	0.507	0.96 (0.64–1.46)	0.861	1.20 (0.87–1.64)	0.263
Donor CMV− (vs. CMV+)	3.16 (1.48–6.75)	0.003	1.78 (1.26–2.51)	0.001	1.07 (0.72–1.60)	0.734	0.91 (0.67–1.24)	0.560
Complex karyotype (no vs. yes)	2.49 (0.97–6.41)	0.058	1.05 (0.69–1.61)	0.818	0.50 (0.28–0.90)	0.021	0.99 (0.85–1.15)	0.884
Donor: M(M)UD vs. matched-RD	1.18 (0.32–4.29)	0.803	1.17 (0.72–1.92)	0.528	2.78 (1.38–5.58)	0.004	0.78 (0.52–1.17)	0.229
Age of donor at allo-HCT (continuous)	1.03 (0.99–1.07)	0.128	1.01 (1.0–1.02)	0.173	1.00 (0.99–1.02)	0.646	0.99 (0.97–1.00)	0.073
MRD positive before allo-HCT (no vs. yes)	0.94 (0.42–2.10)	0.879	1.23 (0.83–1.82)	0.306	1.01 (0.65–1.58)	0.959	0.96 (0.91–1.01)	0.144
In-vivo T-cell depletion (no vs. yes)	0.26 (0.07–0.91)	0.035	1.66 (0.95–2.91)	0.077	1.90 (0.99–3.65)	0.053	1.03 (0.51–2.08)	0.945
Tac vs CSA	5.57 (0.52–59.47)	0.155	1.13 (0.14-8.90)	0.906	0.0000002 (0-Inf)	0.994	3.22 (0.74–13.99)	0.119
Days until first stop of IS (continuous)	1 (1–1)	0.644	1 (1–1)	0.333	1 (1–1)	0.851	1 (1–1)	0.783
IS at day +180 (yes vs. no)	0.80 (0.35–1.83)	0.592	1.10 (0.73–1.65)	0.646	1.01 (0.65–1.59)	0.95	1.73 (1.19–2.51)	0.004
IS at two years (yes vs. no)	0.41 (0.16–1.03)	0.057	1.12 (0.69–1.84)	0.641	0.60 (0.35–1.01)	0.054	1.05 (0.68–1.62)	0.815

*HR* hazard ratio, *CI* confidence interval, *allo-HCT* allogeneic hematopoietic stem cell transplantation, *AML* acute myelogenous leukemia, *MDS* myelodysplastic syndrome, *ECOG* Eastern Cooperative Oncology Group score, *PS* Performance Score, *HCT-CI* hematopoietic cell transplantation-specific comorbidity index*, CMV* cytomegalovirus*, MUD* matched unrelated donor*, MMUD* mismatched unrelated donor*, matched-RD* matched related donor*, MRD* measurable residual disease, *Tac* Tacrolimus, *CSA* Ciclosporin A, *IS* systemic immunosuppression.

Univariate analysis (Supplementary Table 3) supported these findings, demonstrating additional associations between T-cell reconstitution and patient CMV seropositivity. In-vivo TCD was associated with normalization of CD8+ T and B cells. Higher donor age and higher HCT-CI scores were associated with normalization of CD4+ cells, while patients with earlier CD19+ recovery had higher HCT-CI scores. Application of systemic immunosuppression on day +180 and at 2 years was positively associated with normalization of CD4+ but negatively with normalization of CD8+ T cell counts.

## Humoral immune reconstitution

Humoral immune recovery was assessed through serial immunoglobulin G (IgG) values, with data available for 93% of the original cohort. By day +100, 57% of patients who received FluTreo had normalized IgG levels, compared with 38% in the FluTBI group (*p* = 0.003) (Supplementary Fig. 1d, h). IgG normalization rates increased over time, reaching 95% and 89% at two years in the FluTreo and FluTBI groups, respectively. Multivariate analysis revealed that IgG normalization at two years was significantly associated with FluTreo conditioning, lower HCT-CI scores, and discontinued systemic immunosuppression at day +180 (Table 1, Supplementary Table 3).

## Impact of immune reconstitution on survival outcomes and GvHD

A comparison between the conditioning regimens revealed that NRM was higher in the FluTreo patients, while OS, RFS and incidence of relapse and GvHD were similar between groups (Supplementary Table 4). Subsequently, we investigated how immune reconstitution influences post-transplant outcomes (Supplementary Table 5). Persistent deficiencies in CD19+ lymphocytes by day +180, and IgG at two years were associated with reduced OS and RFS **(**Supplementary Fig. 2a–f). Given that time of cellular and humoral immune reconstitution varies for each patient individually, we performed additional landmark analyses excluding patients with early death, revealing similar associations. Patients who achieved CD3+/CD8+ or CD19+ reconstitution by day +180 or IgG levels at two years displayed lower NRM, whereas early CD3+/CD4+ recovery was associated with higher NRM (Supplementary Fig. 3a–d) with infections as the leading cause (Supplementary Table 6). Moreover, patients without normalization of B cells by day +180 showed a trend towards higher relapse incidences.

Patients who failed to achieve CD3+/CD8+ normalization by day +180 or IgG normalization at two years had higher rates of acute GvHD (Supplementary Fig. 4b, d). Chronic GvHD (cGvHD) was more common among patients who experienced delayed CD19+ normalization by day +180, whereas patients whose CD3+/CD4+ counts were normalized by day +180 had higher rates (Supplementary Fig. 4e, g). Normal IgG levels at two years were associated with reduced CMV/EBV/HSV reactivation (Supplementary Table 5).

Multivariate analysis of normalization of all three lymphocyte subsets by day +180 indicated correlations between CD3+/CD4+ reconstitution and cGvHD as well as CD19+ with OS, RFS, NRM and cGvHD (Supplementary Fig. 5).

## Toxicity profiles across conditioning regimens

Comparison of toxicity profiles between conditioning regimens (Supplementary Table 7) revealed occurrence of high-grade mucositis significantly more often in the FluTBI cohort. Acute and long-term (≥3 months post-transplant) toxicities affecting lungs, liver, skin, or other organs were rare in both cohorts. Reactivations of CMV, Ebstein-Barr or herpes simplex viruses requiring therapy were not significantly different, although numerically higher in FluTBI patients.

By day +100 post-transplant, ECOG PS worsened in the FluTBI cohort relative to baseline (*p* < 0.05), whereas it improved continuously in the FluTreo group (Supplementary Fig. 6).

This study compared two dose-reduced conditioning regimens, FluTreo and FluTBI, focusing on immune reconstitution. The long observation period allowed us to include a meaningful number of patients, but during this time there have been changes in patient management, like the implementation of letermovir prophylaxis for CMV-positive patients in 2018, which might influence our results. Despite limitations due to its retrospective nature, we identified notable differences in lymphocyte and immunoglobulin recovery. Specifically, FluTreo was associated with earlier normalization of CD4+ T-lymphocyte counts and IgG levels, although this cohort was significantly older. Multivariate analysis confirmed FluTBI as a risk factor.

Immune reconstitution is a critical, yet incompletely understood, component of the post-transplant course. TBI-containing protocols have been linked to delayed recovery of CD3+ lymphocytes [3], particularly affecting CD4+ T cells [4]. In contrast, FluTreo showed improved B-cell recovery [5]. Our findings align with these observations: FluTreo was associated with earlier normalization of CD3+/CD4+ cells, as well as improved humoral immunity (i.e. higher IgG-levels at one and two years).

Other factors associated with immune reconstitution included the use of in-vivo TCD, and CMV-seropositivity, supporting the results of previous studies [6, 7].

Corresponding to earlier work, rapid immune reconstitution of T and B cells was associated with improved survival outcomes [8–11], additionally, we found associations with higher IgG values. These observations may help explain previously observed reduction of treatment-related mortality after conditioning with FluTreo [1, 2, 12].

The possible impact of immunosuppressive treatment or the GvHD itself on lymphocyte increase remains speculative. In this analysis, early CD3+/CD4+ T-cell recovery was associated with a higher incidence of cGvHD, while patients with early B-cell normalization displayed less cGvHD mirroring previous findings [10]. Importantly, this retrospective analysis does not clarify whether delayed immune reconstitution is a cause or a consequence of complications like GvHD or infections.

Patients conditioned with TBI experienced more frequent mucositis and slower improvement in ECOG PS than FluTreo-treated patients, suggesting that FluTBI patients might have more early complications leading to slower recovery while FluTreo patients, despite being older, recovered better and potentially experience an earlier regain of autonomy with improved quality of life in the early post-transplant phase.

Taken together, our findings indicate that FluTreo offers several advantages over FluTBI, including enhanced CD4+ T-cell and IgG recovery, and fewer acute toxicities.

## Supplementary information

Supplementary Information

## Data Availability

The original contributions presented in the study are included in the article / supplementary material. Further inquiries can be directed to the corresponding author.
