# Supplementary material for: Immune reconstitution in AML and MDS patients undergoing allogeneic hematopoietic cell transplantation (allo-HCT) with treosulfan- or TBI-based conditioning
Source: Bone Marrow Transplant. 2025 Aug 22;60(11):1533–6. doi: 10.1038/s41409-025-02702-2 (PMC12583118; doi:10.1038/s41409-025-02702-2)
Supplement: Supplementary file 1 — Supplementary Information [file 41409_2025_2702_MOESM1_ESM.docx]

**Supplementary Information**

**Immune Reconstitution in AML and MDS Patients Undergoing Allogeneic Hematopoietic Cell Transplantation (allo-HCT) with Treosulfan- or TBI-based Conditioning**

Lina Kolloch^1*^, Philipp Berning^1*^, Jörn C. Albring^1^, Julian Ronnacker^1^, Christian Reicherts^1^, Simon Call^1^, Julia Marx^1^, Matthias Floeth^1^, Eva Esseling^1^, Hans Theodor Eich^2^, Christoph Schliemann^1^, Jan-Henrik Mikesch^1^, Georg Lenz^1^, Matthias Stelljes^1^

^1^ Department of Hematology and Oncology, University Hospital Muenster, Muenster, Germany

^2^ Department of Radiation Oncology, University Hospital Muenster, Muenster, Germany

^*^ These authors contributed equally to this work

Corresponding author: Matthias Stelljes, Department of Hematology and Oncology, University Hospital Muenster, Muenster, Germany, Phone: +492518352801, Email: [stelljes@uni-muenster.de](mailto:stelljes@uni-muenster.de).

# Methods

*Data Collection*

We retrospectively analyzed toxicity profiles and cellular and humoral immune reconstitution in a cohort of 311 patients with acute myeloid leukemia (AML) or myelodysplastic syndrome (MDS) who underwent their first allogeneic hematopoietic cell transplantation (allo-HCT) at the University Hospital Münster, Germany, between 2011 and 2022. AML patients were transplanted in complete remission. The conditioning regimen consisted of either fludarabine (120 mg/m^2^) plus fractionated total-body-irradiation (4 x 2 Gy) or fludarabine (150 mg/m^2^) plus treosulfan (30 g/m^2^ or 42 g/m^2^). Further details on these conditioning protocols and the course of allo-HCT have been published elsewhere (1). All data were obtained from electronic patient records. The local ethics committee approved the study. Informed consent was obtained from all patients prior to transplantation, and no patient explicitly refused the use of their data. All study procedures were conducted in accordance with the Declaration of Helsinki, relevant guidelines, and local regulations.

*Infection prophylaxis and monitoring*

Infection prophylaxis and monitoring during conditioning and after allo-HCT was conducted similarly for all patients, independent from their conditioning. Patients received antimycotic prophylaxis from day -1 until at least day +70. Aciclovir was started on day -1 and continued for at least one year, until the termination of immunosuppression and until the recovery of CD4+ T lymphocytes. Ideally, patients were vaccinated against the herpes zoster virus before stopping aciclovir. Prophylaxis against Pneumocystis jirovecii commenced before the start of conditioning and continued for at least one year, until the termination of immunosuppression, and until the recovery of CD4+ T lymphocytes. From 2018, cytomegalovirus (CMV)-positive patients received Letermovir at least until d+100. In case of CMV-/ Ebstein-Barr virus (EBV)-positive patients or donors, CMV and EBV copies were monitored at least twice, respectively once a week in the first two month and then at least biweekly until the end of immunosuppression and recovery of CD4+ T lymphocytes. If CMV or EBV copies increased, patients were monitored more closely, and therapy was started for CMV copies >1000 IU/ml and EBV copies >500,000 IU/ml. Toxoplasmosis-positive patients were monitored for toxoplasmosis every two weeks until the end of immunosuppression. All prophylaxes were adjusted according to organ function, tolerance, and additional medication.

*Definitions*

AML was classified according to the European LeukemiaNet (ELN) 2017 guidelines (2), and MDS were categorized using the Revised International Prognostic Scoring System (IPSS-R) (3). Overall survival (OS) and relapse-free survival (RFS) were calculated from the date of transplantation. Patients were censored at their last follow-up if they had not experienced an event. Morphologic (overt) relapse was defined as a cytologic or flow cytometric blast count ≥5% in the bone marrow or peripheral blood and / or extramedullary disease confirmed by histopathological analysis. For OS, death from any cause was considered an event. RFS was defined as survival without morphologic relapse. Non-relapse mortality (NRM) was defined as any death occurring in the absence of documented AML or MDS relapse. For grading of toxicities, the common toxicity criteria for adverse events (CTCAE) version 4 (v4) was used.

*Measurement of Lymphocyte Subpopulations and IgG*

Lymphocyte subpopulations and serum IgG levels were measured at the University Hospital Münster. For determination of lymphocyte subpopulations, cells from ethylene diamine tetraacetic acid (EDTA) blood were stained with fluorescently labeled antibodies binding to the specific lymphocyte marker (CD3, CD4, CD8, CD19) and analyzed via flow cytometry on a FACSCanto II. The following reference ranges were used: CD3+ 700–2100/µl, CD3+/CD4+ 300–1400/µl, CD3+/CD8+ 200–900/µl, CD19+ 100–500/µl, and IgG 700–1600 mg/dl. Values were considered “normalized” if they were ≥ the lower limit of the respective reference range. Lymphocyte subpopulations were assessed on days +60 (±15), +100 (±20), +180 (±30) and +360 (±60) post-transplant, while IgG levels were measured on days +100 (±20), +360 (±60), and +720 (±100) post-transplant.

*Statistical Analysis*

All statistical analyses were conducted using R-software (version 4.2.3; http://www.R-project.org). OS and RFS were calculated from the date of transplantation using the Kaplan-Meier method, with patients censored at their last follow-up visit. Differences in OS and RFS were assessed using the log-rank test, and cumulative incidences were compared with Gray’s Test. Toxicities were evaluated using the Chi-square test, whereas the Man-Whitney U and Wilcoxon test were applied for assessing ECOG performance status (PS). Comparisons of lymphocyte subpopulations and IgG values were conducted via Student’s t-test and Chi-square test as appropriate. Multivariable analysis was performed using a Cox proportional hazards model, yielding hazard ratios (HR) with 95% confidence intervals (CI). All statistical tests were two-sided, and p-values < 0.05 were considered statistically significant.

**Suppl. Figure 1. Cellular and humoral immune reconstitution by conditioning regimen**

a) b) c) d)

**
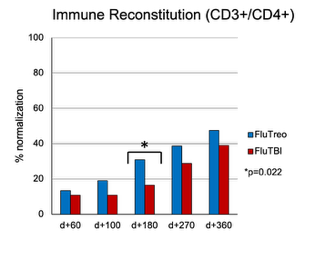

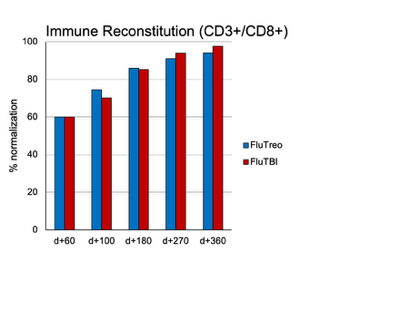

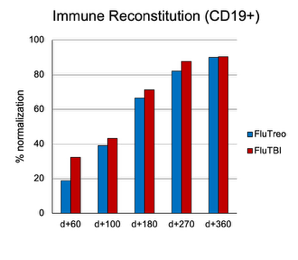

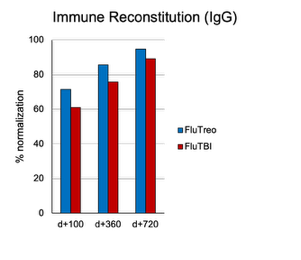
**

e) f) g) h)

**
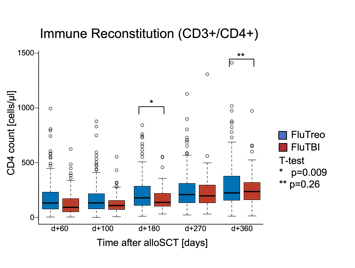

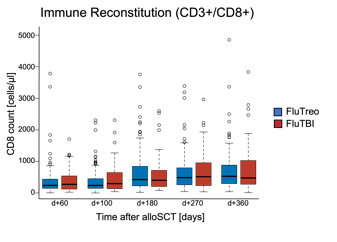

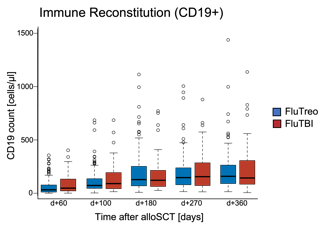

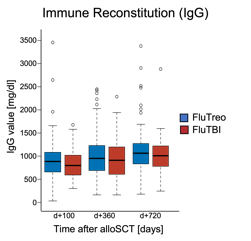
**

The percentage of patients with normalized lymphocyte/ IgG levels (**a-d**) and raw lymphocyte counts/ IgG levels (**e-f**) for the FluTreo and the FluTBI group are presented at different time points after allo-HCT as indicated. **a/e**) CD3+/CD4+ lymphocytes, **b/f**) CD3+/CD8+ lymphocytes, **c/g**) CD19+ lymphocytes, **d/h**) IgG levels.

**Suppl. Figure 2. Kaplan-Meier estimates by CD19+/ IgG reconstitution after allo-HCT**

a) b)

c) d)

e) f)

**
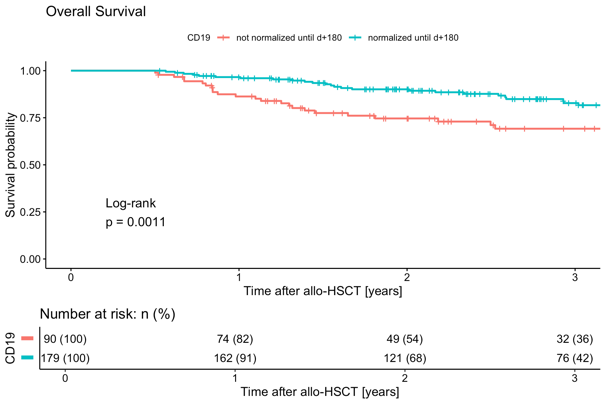

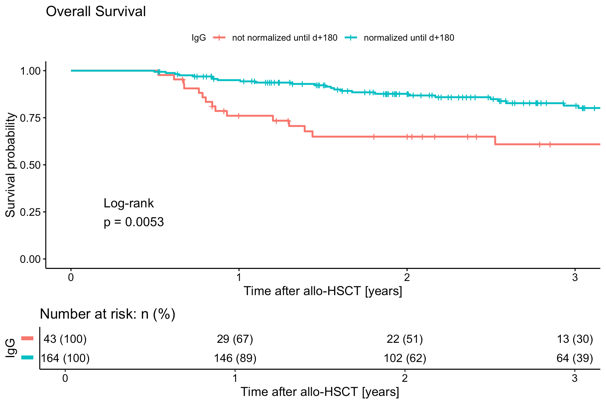
**

**a-d**: Overall survival (**a, c**) and relapse-free survival (**b, d**) for patients with a survival of more than 60 days with or without normalization of CD19+ lymphocytes by day +180 after allo-HCT (**a, b**) and patients with or without normalization of IgG levels at two years after allo-HCT (**c, d**).

**e/f**: Landmark analysis with overall survival for patients with a survival of more than 180 days with or without normalization of CD19+ lymphocytes (**e**) or IgG levels (**f**) by day +180 after allo-HCT.

**Suppl. Figure 3. Cumulative incidences of NRM by normalization of lymphocyte subpopulations by 180 days/ IgG levels by two years after allo-HCT**

a) b)

Non-Relapse Mortality, CD4+, p=0.007 Non-Relapse Mortality, CD8+, p=0.002

**
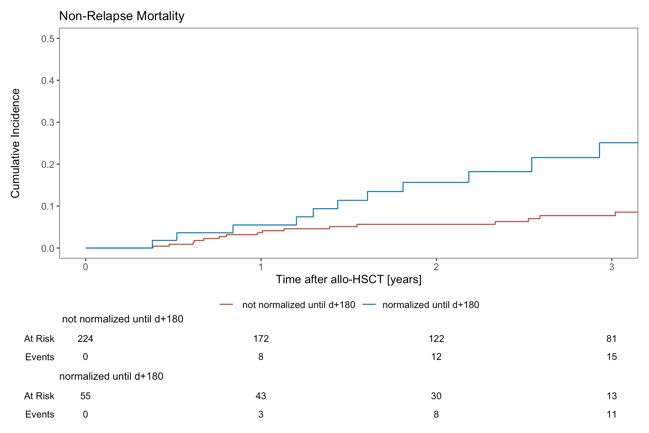
**  **
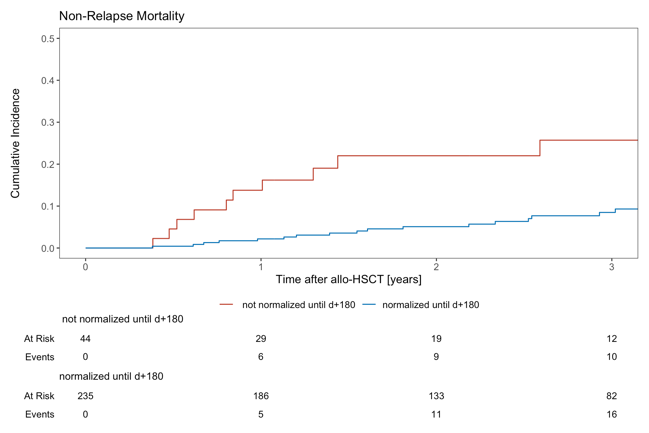
**

c) d)

Non-Relapse Mortality, CD19+, p=0.031 Non-Relapse Mortality, IgG, p<0.001


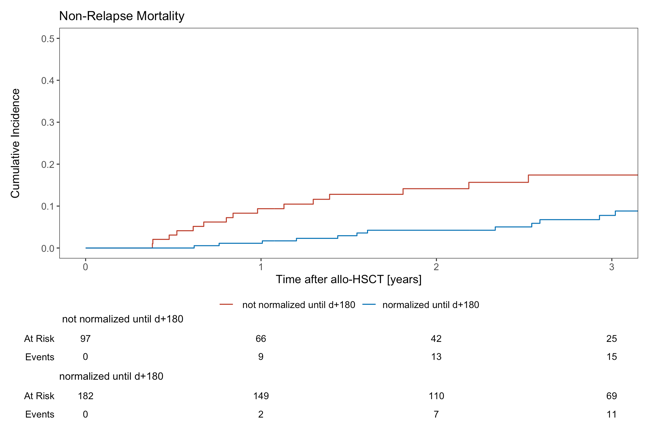
 **
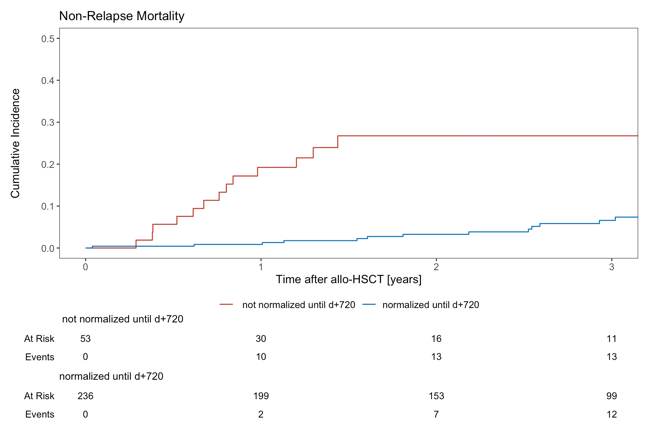
**

Cumulative incidences of non-relapse mortality for patients with a survival of more than 60 days with or without normalization of CD3+/CD4+ (**a**), CD3+/CD8+ (**b**), CD19+ (**c**) lymphocytes by day +180 after allo-HCT and IgG levels by two years after allo-HCT (**d**).

**Suppl. Figure 4. Cumulative incidences of acute and chronic GvHD by normalization of lymphocyte subpopulations by 180 days/ IgG levels by two years after allo-HCT**

a) b)

acute GvHD grade II-IV, CD4+, p=0.14 acute GvHD grade II-IV, CD8+, p=0.006


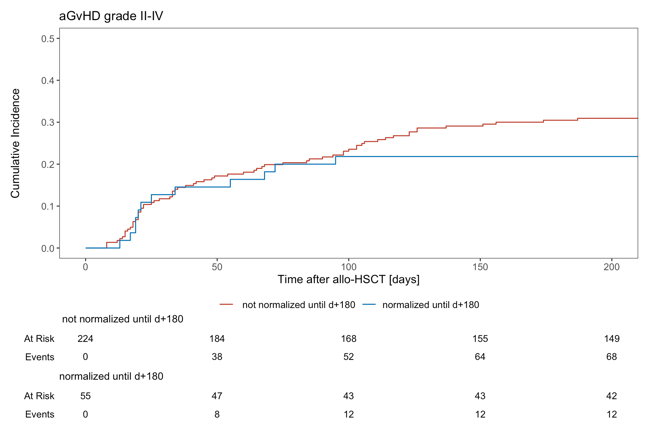

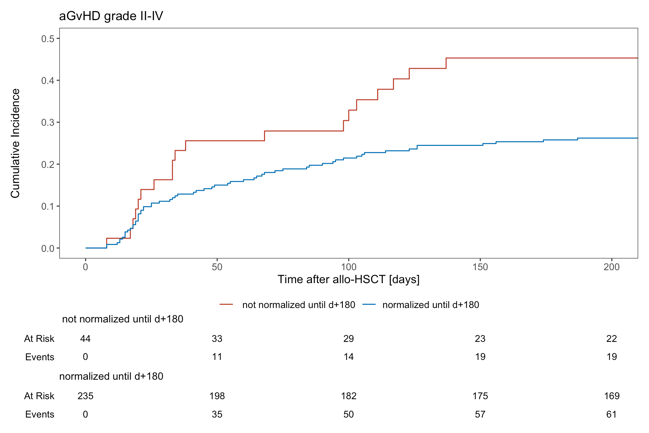


c) d)

acute GvHD grade II-IV, CD19+, p=0.2 acute GvHD grade II-IV, IgG, p<0.001

**
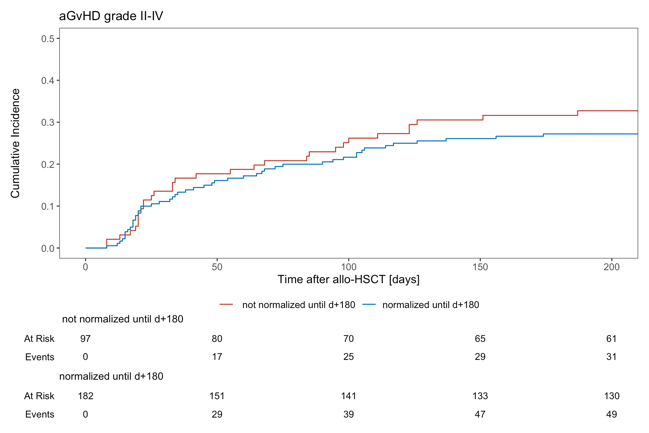
** **
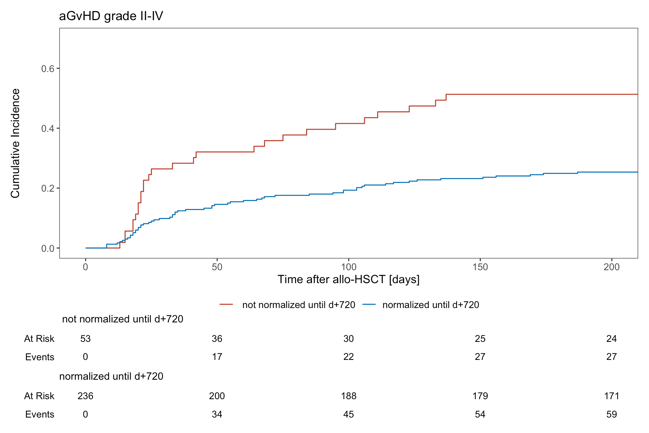
**

e) f)

chronic GvHD, CD4+, p<0.001 chronic GvHD, CD8+, p=0.6

**
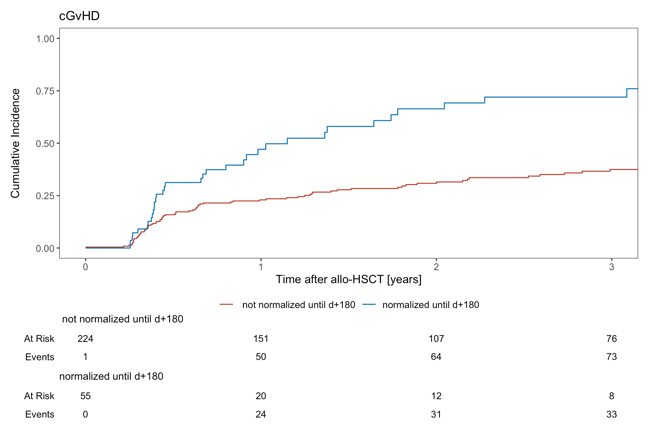
**  **
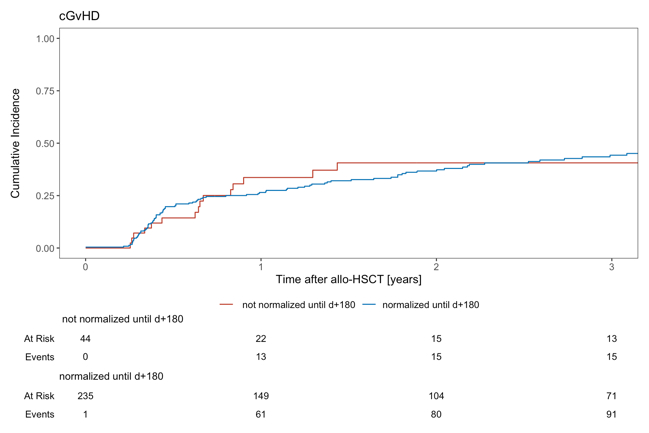
**

g) h)

chronic GvHD, CD19+, p=0.007 chronic GvHD, IgG, p=0.4

**
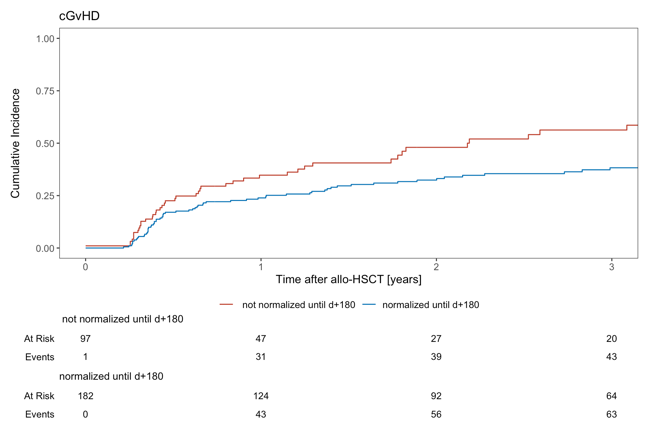

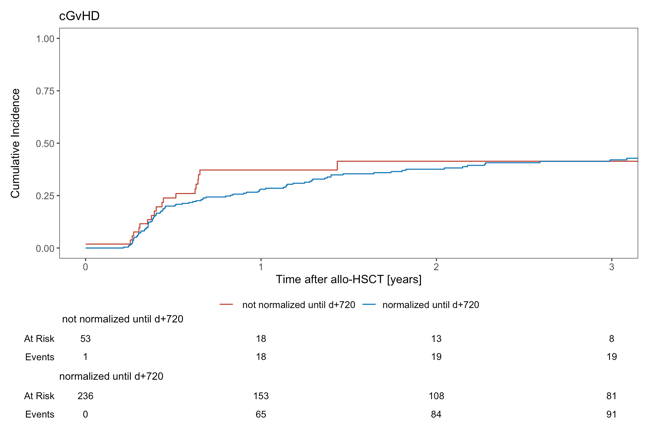
**

**a-d**: Cumulative incidences of acute GvHD for patients with a survival of more than 60 days with or without normalization of CD3+/CD4+ (**a**), CD3+/CD8+ (**b**), CD19+ (**c**) lymphocytes by day +180 after allo-HCT and IgG levels by two years after allo-HCT (**d**). **e-h**: Cumulative incidences of chronic GvHD for patients with or without normalization of CD3+/CD4+ (**e**), CD3+/CD8+ (**f**), CD19+ (**g**) lymphocytes by day +180 after allo-HCT and IgG values by two years after allo-HCT (**h**).

**Suppl. Figure 5. Forest plot for multivariate outcomes associated with cellular immune reconstitution by day +180 after allo-HCT**


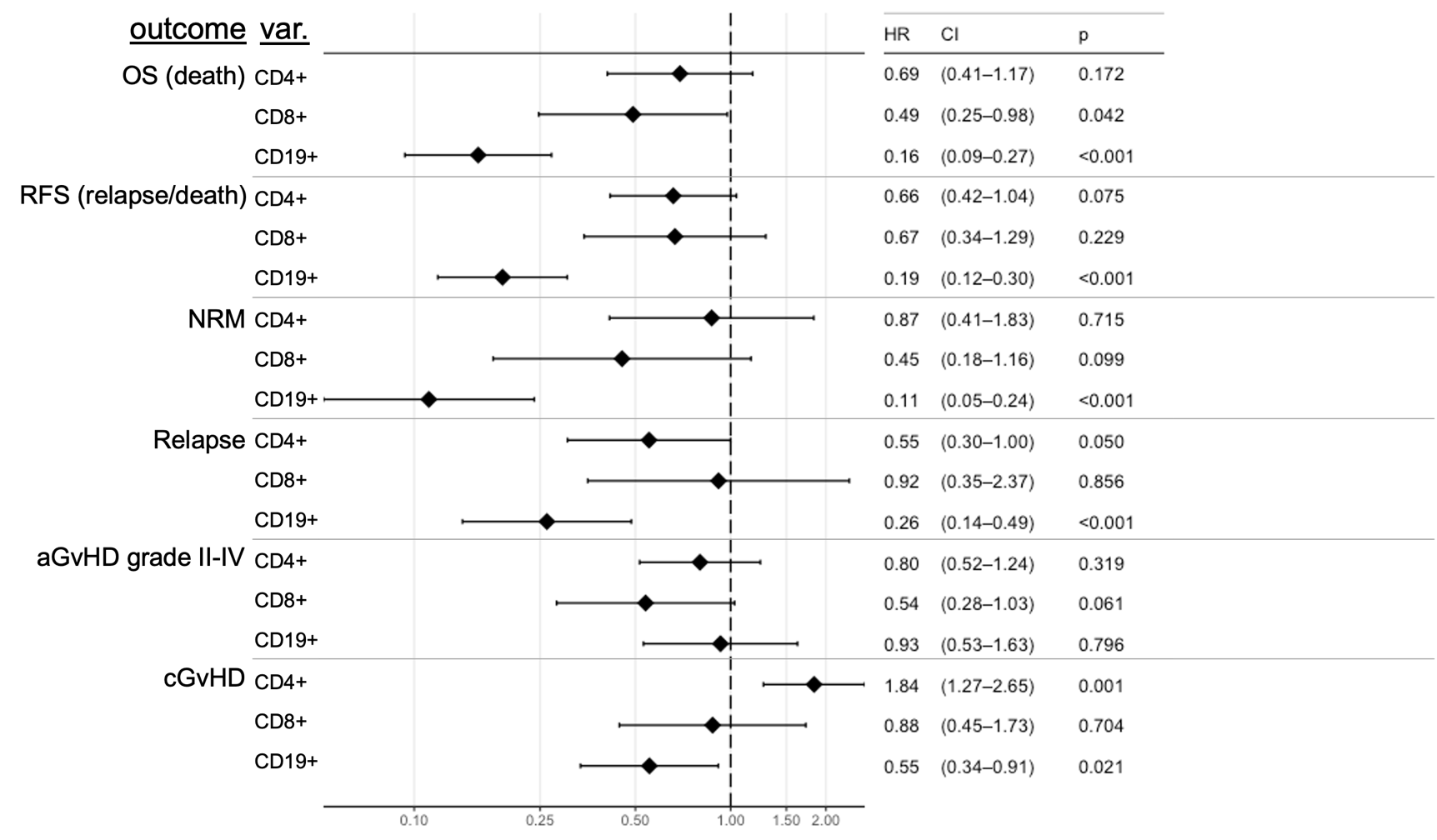


Hazard ratios (HR) for different outcomes after allo-HCT are shown with a 95% confidence interval (CI). As influencing parameters normalization of CD3+/CD4+, CD3+/CD8+ and CD19+ lymphocytes by day +180 after allo-HCT are considered.

**Suppl. Figure 6. ECOG performance score by conditioning regimen**

a)

**
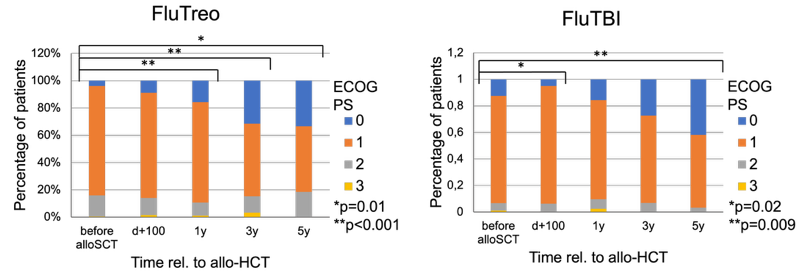
**

Percentage of patients with the respective ECOG performance score (PS) for the indicated time points prior and after allo-HCT by conditioning regimen (FluTreo vs. FluTBI).

**Suppl. Table 1. Baseline characteristics by conditioning regimen fludarabin/treosulfan vs. fludarabin/TBI**

| **Transplant characteristics** | **FluTreo** | **FluTBI** | **P value** |
| --- | --- | --- | --- |
|  | (N=207) | (N=104) |  |
| **Age at allo-HCT [years]** |  |  | < 0.001 |
| Median [Min, Max] | 64.0 [19.0, 76.0] | 47.0 [18.0, 69.0] |  |
| **Age at allo-HCT over 60 years** |  |  | < 0.001 |
| No | 69 (33.3%) | 96 (92.3%) |  |
| Yes | 138 (66.7%) | 8 (7.7%) |  |
| **Sex** |  |  | 0.44 |
| Female | 84 (40.6%) | 47 (45.2%) |  |
| Male | 123 (59.4%) | 57 (54.8%) |  |
| **Diagnosis** |  |  | < 0.001 |
| AML | 123 (59.4%) | 92 (88.5%) |  |
| MDS | 84 (40.6%) | 12 (11.5%) |  |
| **HCT-CI Score grouped** |  |  | < 0.001 |
| 0-2 | 104 (50.2%) | 75 (72.1%) |  |
| 3-5 | 80 (38.6%) | 23 (22.1%) |  |
| >5 | 23 (11.1%) | 6 (5.8%) |  |
| **ECOG PS** |  |  | 0.002 |
| 0 | 8 (3.9%) | 13 (12.5%) |  |
| 1 | 166 (80.2%) | 84 (80.8%) |  |
| 2 | 32 (15.5%) | 6 (5.8%) |  |
| 3 | 1 (0.5%) | 1 (1.0%) |  |
| **Complex karyotype** |  |  |  |
| No | 163 (78.7%) | 91 (87.5%) | 0.023 |
| Yes | 42 (20.3%) | 10 (9.6%) |  |
| Missing | 2 | 3 |  |
| **Donor type** |  |  | 0.046 |
| Matched-related | 50 (24.2%) | 15 (14.4%) |  |
| 10/10 HLA-matched unrelated | 132 (63.8%) | 68 (65.4%) |  |
| 9/10 HLA-matched unrelated | 25 (12.1%) | 21 (20.2%) |  |
| **Age of donor at allo-HCT** |  |  | 0.005 |
|  | 34 [18, 72] | 31 [19, 61] |  |
| ***In-vivo* T-cell depletion** |  |  |  |
| No | 47 (22.7%) | 13 (12.5%) |  |
| Yes | 160 (77.3%) | 91 (87.5%) |  |
| **GvHD prevention** |  |  |  |
| Cyclosporin A + MTX / MMF | 205 (99.0%) | 104 (100%) | 0.553 |
| Tacrolimus + MTX / MMF | 2 (1.0%) | 0 (0%) |  |
| **ELN2017 risk classification** |  |  | 0.011 |
| Favorable | 28 (22.8%) | 30 (32.6%) |  |
| Intermediate | 43 (35.0%) | 41 (44.6%) |  |
| Adverse | 52 (42.3%) | 21 (22.8%) |  |
| MDS patient | 84 | 12 |  |
| **IPSSR risk classification** |  |  | 0.837 |
| Low risk | 4 (4.8%) | 0 (0%) |  |
| Intermediate risk | 17 (20.2%) | 3 (25.0%) |  |
| High risk | 63 (75.0%) | 9 (75.0%) |  |
| AML patient | 123 | 92 |  |
| **Days until first stop of IS** |  |  | 0.41 |
| Median [Min, Max] | 127 [11, 1490] | 127 [33, 2320] |  |
| **IS at day +180 (yes vs. no)** |  |  | 0.629 |
| Yes | 141 (68.1%) | 68 (65.4%) |  |
| No | 66 (31.9%) | 36 (34.6%) |  |
| **IS at 2 years after allo-HCT** |  |  | 0.574 |
| Yes | 172 (83.1%) | 89 (85.6%) |  |
| No | 35 (16.9%) | 15 (14.4%) |  |

*FluTreo* fludarabine/treosulfan, *FluTBI* fludarabine/8Gy total body irradiation/fludarabine, *allo-HCT* allogeneic hematopoietic stem cell transplantation, *AML* acute myelogenous leukemia, *MDS* myelodysplastic symdroma, *HCT-CI* hematopoietic cell transplantation-specific comorbidity index*, ECOG* Eastern Cooperative Oncology Group, *PS* Performance Score, *HLA* human leukocyte antigens, *GvHD* graft-versus-host disease*, MTX* methotrexate*, MMF* mycophenolate mofetil, *ELN2017* European Leukemia Net 2017 classification, *IPSSR* Revised International Prognostic Scong System for myelodysplastic syndromes risk assessment, *IS* systemic immunosuppression.

**Suppl. Table 2. Baseline Characteristics by CD3+/4+, CD3+/8+ and CD19+ lymphocyte reconstitution by day +180 and IgG normalization by two years after allo-HCT**

|  | **CD3+/CD4+ lymphocytes**  **normalized by day +180** | | **CD3+/CD8+ lymphocytes normalized by day +180** | | **CD19+ lymphocytes normalized by day +180** | | **IgG normalized by two years** | |
| --- | --- | --- | --- | --- | --- | --- | --- | --- |
|  | no | yes | no | yes | no | yes | no | yes |
|  | (N=224) | (N=55) | (N=44) | (N=235) | (N=97) | (N=182) | (N=53) | (N=236) |
| **Age at allo-HCT [years]** |  |  |  |  |  |  |  |  |
| Median [Min, Max] | 59.0 [18.0, 76.0] | 61.0 [33.0, 73.0] | 59.0 [21.0, 76.0] | 60.0 [18.0, 74.0] | 60.0 [22.0, 74.0] | 60.0 [18.0, 76.0] | 59.0 [27.0, 76.0] | 59.0 [18.0, 74.0] |
| **Age at allo-HCT over 60 years** |  |  |  |  |  |  |  |  |
| No | 117 (52.2%) | 27 (49.1%) | 25 (56.8%) | 119 (50.6%) | 52 (53.6%) | 92 (50.5%) | 33 (62.3%) | 124 (52.5%) |
| Yes | 107 (47.8%) | 28 (50.9%) | 19 (43.2%) | 116 (49.4%) | 45 (46.4%) | 90 (49.5%) | 20 (37.7%) | 112 (47.5%) |
| **Conditioning regimen** |  |  |  |  |  |  |  |  |
| FluTreo | 151 (67.4%) | 46 (83.6%) | 31 (70.5%) | 166 (70.6%) | 72 (74.2%) | 125 (68.7%) | 31 (58.5%) | 162 (68.6%) |
| FluTBI | 73 (32.6%) | 9 (16.4%) | 13 (29.5%) | 69 (29.4%) | 25 (25.8%) | 57 (31.3%) | 22 (41.5%) | 74 (31.4%) |
| **Sex** |  |  |  |  |  |  |  |  |
| Female | 94 (42.0%) | 23 (41.8%) | 15 (34.1%) | 102 (43.4%) | 45 (46.4%) | 72 (39.6%) | 22 (41.5%) | 101 (42.8%) |
| Male | 130 (58.0%) | 32 (58.2%) | 29 (65.9%) | 133 (56.6%) | 52 (53.6%) | 110 (60.4%) | 31 (58.5%) | 135 (57.2%) |
| **Diagnosis** |  |  |  |  |  |  |  |  |
| AML | 150 (67.0%) | 39 (70.9%) | 26 (59.1%) | 163 (69.4%) | 60 (61.9%) | 129 (70.9%) | 41 (77.4%) | 161 (68.2%) |
| MDS | 74 (33.0%) | 16 (29.1%) | 18 (40.9%) | 72 (30.6%) | 37 (38.1%) | 53 (29.1%) | 12 (22.6%) | 75 (31.8%) |
| **HCT-CI Score grouped** |  |  |  |  |  |  |  |  |
| 0-2 | 136 (60.7%) | 23 (41.8%) | 20 (45.5%) | 139 (59.1%) | 45 (46.4%) | 114 (62.6%) | 26 (49.1%) | 141 (59.7%) |
| 3-5 | 71 (31.7%) | 26 (47.3%) | 18 (40.9%) | 79 (33.6%) | 39 (40.2%) | 58 (31.9%) | 20 (37.7%) | 78 (33.1%) |
| >5 | 17 (7.6%) | 6 (10.9%) | 6 (13.6%) | 17 (7.2%) | 13 (13.4%) | 10 (5.5%) | 7 (13.2%) | 17 (7.2%) |
| **CMV-status patient** |  |  |  |  |  |  |  |  |
| No | 94 (42.0%) | 12 (21.8%) | 23 (52.3%) | 83 (35.3%) | 39 (40.2%) | 67 (36.8%) | 23 (43.4%) | 86 (36.4%) |
| Yes | 127 (56.7%) | 42 (76.4%) | 21 (47.7%) | 148 (63.0%) | 56 (57.7%) | 113 (62.1%) | 28 (52.8%) | 148 (62.7%) |
| Missing | 3 (1.3%) | 1 (1.8%) | 0 (0%) | 4 (1.7%) | 2 (2.1%) | 2 (1.1%) | 2 (3.8%) | 2 (0.8%) |
| **CMV-status donor** |  |  |  |  |  |  |  |  |
| No | 123 (54.9%) | 20 (36.4%) | 34 (77.3%) | 109 (46.4%) | 48 (49.5%) | 95 (52.2%) | 24 (45.3%) | 123 (52.1%) |
| Yes | 100 (44.6%) | 34 (61.8%) | 10 (22.7%) | 124 (52.8%) | 48 (49.5%) | 86 (47.3%) | 26 (49.1%) | 112 (47.5%) |
| Missing | 1 (0.4%) | 1 (1.8%) | 0 (0%) | 2 (0.9%) | 1 (1.0%) | 1 (0.5%) | 3 (5.7%) | 1 (0.4%) |
| **ECOG PS** |  |  |  |  |  |  |  |  |
| 0 | 13 (5.8%) | 5 (9.1%) | 1 (2.3%) | 17 (7.2%) | 7 (7.2%) | 11 (6.0%) | 4 (7.5%) | 16 (6.8%) |
| 1 | 184 (82.1%) | 42 (76.4%) | 32 (72.7%) | 194 (82.6%) | 74 (76.3%) | 152 (83.5%) | 45 (84.9%) | 190 (80.5%) |
| 2 | 25 (11.2%) | 8 (14.5%) | 11 (25.0%) | 22 (9.4%) | 14 (14.4%) | 19 (10.4%) | 4 (7.5%) | 29 (12.3%) |
| 3 | 2 (0.9%) | 0 (0%) | 0 (0%) | 2 (0.9%) | 2 (2.1%) | 0 (0%) | 0 (0%) | 1 (0.4%) |
| **Complex karyotype** |  |  |  |  |  |  |  |  |
| No | 181 (80.8%) | 47 (85.5%) | 33 (75.0%) | 195 (83.0%) | 72 (74.2%) | 156 (85.7%) | 45 (84.9%) | 196 (83.1%) |
| Yes | 39 (17.4%) | 8 (14.5%) | 11 (25.0%) | 36 (15.3%) | 24 (24.7%) | 23 (12.6%) | 7 (13.2%) | 37 (15.7%) |
| Missing | 4 (1.8%) | 0 (0%) | 0 (0%) | 4 (1.7%) | 1 (1.0%) | 3 (1.6%) | 1 (1.9%) | 3 (1.3%) |
| **Donor type** |  |  |  |  |  |  |  |  |
| Matched-related | 28 (12.5%) | 34 (61.8%) | 15 (34.1%) | 47 (20.0%) | 29 (29.9%) | 33 (18.1%) | 18 (34.0%) | 46 (19.5%) |
| 10/10 HLA-matched unrelated | 164 (73.2%) | 18 (32.7%) | 21 (47.7%) | 161 (68.5%) | 47 (48.5%) | 135 (74.2%) | 28 (52.8%) | 156 (66.1%) |
| 9/10 HLA-matched unrelated | 32 (14.3%) | 3 (5.5%) | 8 (18.2%) | 27 (11.5%) | 21 (21.6%) | 14 (7.7%) | 7 (13.2%) | 34 (14.4%) |
| **Age of donor at allo-HCT** |  |  |  |  |  |  |  |  |
| Median [Min, Max] | 32 [18, 72] | 54 [18, 71] | 34 [19, 71] | 33 [18, 72] | 35 [19, 71] | 33 [18, 72] | 35 [18, 71] | 33 [18, 72] |
| **MRD status before allo-HCT** |  |  |  |  |  |  |  |  |
| No | 64 (28.6%) | 15 (27.3%) | 19 (43.2%) | 60 (25.5%) | 27 (27.8%) | 52 (28.6%) | 17 (32.1%) | 69 (29.2%) |
| Yes | 130 (58.0%) | 34 (61.8%) | 20 (45.5%) | 144 (61.3%) | 56 (57.7%) | 108 (59.3%) | 28 (52.8%) | 139 (58.9%) |
| No marker | 9 (4.0%) | 2 (3.6%) | 1 (2.3%) | 10 (4.3%) | 2 (2.1%) | 9 (4.9%) | 4 (7.5%) | 8 (3.4%) |
| Not analyzed | 21 (9.4%) | 4 (7.3%) | 4 (9.1%) | 21 (8.9%) | 12 (12.4%) | 13 (7.1%) | 4 (7.5%) | 20 (8.5%) |
| **Year of transplatation** |  |  |  |  |  |  |  |  |
| Median [Min, Max] | 2020 [2011, 2022] | 2019 [2017, 2022] | 2020 [2015, 2022] | 2019 [2011, 2022] | 2019 {2011, 2022] | 2020 {2012, 2022& | 2019 [2011, 2022] | 2019 [2012, 2022] |
| **Median follow-up [months]** |  |  |  |  |  |  |  |  |
| Median [Min, Max] | 30.2 [0.395, 124] | 27.0 [4.57, 62.2] | 25.1 [2.89, 81.5] | 30.0 [0.395, 124] | 24.0 [2.53, 124] | 31.3 [0.395, 117] | 15.6 [2.89, 89.1] | 33.0 [0.395, 124] |
| **Median Time-to-transplant [months]** |  |  |  |  |  |  |  |  |
| Median [Min, Max] | 3.88 [0, 112] | 3.62 [1.94, 86.1] | 3.73 [0, 112] | 3.85 [1.51, 98.5] | 3.72 [1.78, 83.0] | 3.90 [0, 112] | 3.95 [1.94, 89.0] | 3.87 [0, 112] |
| ***In-vivo* T-cell depletion** |  |  |  |  |  |  |  |  |
| No | 24 (10.7%) | 34 (61.8%) | 14 (31.8%) | 44 (18.7%) | 28 (28.9%) | 30 (16.5%) | 17 (32.1%) | 43 (18.2%) |
| Yes | 200 (89.3%) | 21 (38.2%) | 30 (68.2%) | 191 (81.3%) | 69 (71.1%) | 152 (83.5%) | 36 (67.9%) | 193 (81.8%) |
| **GvHD prevention** |  |  |  |  |  |  |  |  |
| Cyclosporin A + MTX / MMF | 223 (99.6%) | 54 (98.2%) | 43 (97.7%) | 234 (99.6%) | 95 (97.9%) | 182 (100%) | 53 (100%) | 234 (99.2%) |
| Tacrolimus + MTX / MMF | 1 (0.4%) | 1 (1.8%) | 1 (2.3%) | 1 (0.4%) | 2 (2.1%) | 0 (0%) | 0 (0%) | 2 (0.8%) |
| **ELN2017 risk classification** |  |  |  |  |  |  |  |  |
| Favorable | 43 (19.2%) | 8 (14.5%) | 8 (18.2%) | 43 (18.3%) | 12 (12.4%) | 39 (21.4%) | 6 (11.3%) | 50 (21.2%) |
| Intermediate | 61 (27.2%) | 14 (25.5%) | 10 (22.7%) | 65 (27.7%) | 23 (23.7%) | 52 (28.6%) | 15 (28.3%) | 61 (25.8%) |
| Adverse | 47 (21.0%) | 17 (30.9%) | 8 (18.2%) | 56 (23.8%) | 25 (25.8%) | 39 (21.4%) | 20 (37.7%) | 51 (21.6%) |
| MDS patient | 73 (32.6%) | 16 (29.1%) | 18 (40.9%) | 71 (30.2%) | 37 (38.1%) | 52 (28.6%) | 12 (22.6%) | 74 (31.4%) |
| **IPSSR risk classification** |  |  |  |  |  |  |  |  |
| Low risk | 4 (1.8%) | 0 (0%) | 0 (0%) | 4 (1.7%) | 3 (3.1%) | 1 (0.5%) | 0 (0%) | 4 (1.7%) |
| Intermediate risk | 18 (8.0%) | 2 (3.6%) | 2 (4.5%) | 18 (7.7%) | 5 (5.2%) | 15 (8.2%) | 2 (3.8%) | 17 (7.2%) |
| High risk | 52 (23.2%) | 14 (25.5%) | 16 (36.4%) | 50 (21.3%) | 29 (29.9%) | 37 (20.3%) | 10 (18.9%) | 54 (22.9%) |
| AML patient | 150 (67.0%) | 39 (70.9%) | 26 (59.1%) | 163 (69.4%) | 60 (61.9%) | 129 (70.9%) | 41 (77.4%) | 161 (68.2%) |
| **Days until first stop of IS** |  |  |  |  |  |  |  |  |
| Median [Min, Max] | 127 [77, 2320] | 141 [66, 1490] | 153 [92, 946] | 127 [66, 2320] | 139 [77, 1560] | 126 [66, 2320] | 149 [74, 1490] | 126 [66, 2320] |
| **IS at day +180 (yes vs. no)** |  |  |  | 0.02005 |  | 0.1512 |  | 0.283 |
| Yes | 157 (70.1%) | 31 (56.4%) | 23 (52.3%) | 165 (70.2%) | 60 (61.9%) | 128 (70.3%) | 24 (45.3%) | 167 (70.8%) |
| No | 67 (29.9%) | 24 (43.6%) | 21 (47.7%) | 70 (29.8%) | 37 (38.1%) | 54 (29.7%) | 29 (54.7%) | 69 (29.2%) |
| **IS at 2 years after allo-HCT** |  |  |  |  |  |  |  |  |
| Yes | 193 (86.2%) | 41 (74.5%) | 34 (77.3%) | 200 (85.1%) | 85 (87.6%) | 149 (81.9%) | 42 (79.2%) | 199 (84.3%) |
| No | 31 (13.8%) | 14 (25.5%) | 10 (22.7%) | 35 (14.9%) | 12 (12.4%) | 33 (18.1%) | 11 (20.8%) | 37 (15.7%) |

*FluTreo* fludarabine/treosulfan, *FluTBI* fludarabine/8Gy total body irradiation/fludarabine, *allo-HCT* allogeneic hematopoietic stem cell transplantation, *AML* acute myelogenous leukemia, *MDS* myelodysplastic syndrome, *HCT-CI* hematopoietic cell transplantation-specific comorbidity index*, CMV* cytomegalovirus*, ECOG* Eastern Cooperative Oncology Group score, *PS* Performance Score, *HLA* human leukocyte antigens, *MRD* measurable residual disease, *GvHD* graft-versus-host disease*, MTX* methotrexate*, MMF* mycophenolate mofetil, *ELN2017* European Leukemia Net 2017 classification, *IPSSR* Revised International Prognostic Scoring System for myelodysplastic syndromes risk assessment, *IS* systemic immunosuppression.

**Suppl. Table 3. Univariate analysis of baseline characteristics for normalization of lymphocyte subpopulations by day +180/ IgG levels by two years after allo-HCT**

|  | **CD3+/CD4+ lymphocytes**  **normalized until d+180** | | **CD3+/CD8+ lymphocytes normalized until d+180** | | **CD19+ lymphocytes normalized until d+180** | | **IgG normalized until d+720** | |
| --- | --- | --- | --- | --- | --- | --- | --- | --- |
|  | **HR (95% CI for HR)** | **p-value** | **HR (95% CI for HR)** | **p-value** | **HR (95% CI for HR)** | **p-value** | **HR (95% CI for HR)** | **P-value** |
| **Sex female vs. male** | 1 (0.58-1.7) | 1 | 0.82 (0.64-1.1) | 0.14 | 1.1 (0.82-1.5) | 0.51 | 1.1 (0.86-1.4) | 0.39 |
| **FluTBI vs. FluTreo** | 2.3 (1.1-4.8) | 0.021 | 1.1 (0.81-1.4) | 0.6 | 0.87 (0.63-1.2) | 0.38 | 1.5 (1.1-1.9) | 0.0081 |
| **Age before allo-HCT** | 1 (1-1) | 0.1 | 1 (0.99-1) | 0.84 | 0.99 (0.98-1) | 0.21 | 1 (1-1) | 0.08 |
| **Age>60 yrs before allo-HCT** | 1.1 (0.65-1.9) | 0.71 | 1.1 (0.88-1.5) | 0.34 | 0.99 (0.74-1.3) | 0.94 | 1.3 (1-1.7) | 0.052 |
| **Diagnosis AML vs. MDS** | 0.83 (0.47-1.5) | 0.54 | 0.82 (0.62-1.1) | 0.16 | 0.79 (0.57-1.1) | 0.14 | 1.2 (0.94-1.6) | 0.14 |
| **ECOG PS (0-1 vs. >1)** | 1.3 (0.6-2.7) | 0.53 | 0.58 (0.38-0.88) | 0.011 | 0.73 (0.46-1.2) | 0.2 | 1.4 (0.92-2) | 0.12 |
| **HCT-CI score grouped (0-2, 3-5, >5)** | 1.6 (1.1-2.3) | 0.015 | 0.85 (0.69-1) | 0.11 | 0.76 (0.6-0.97) | 0.025 | 0.85 (0.69-1) | 0.11 |
| **Patient CMV- (vs. CMV+)** | 2.3 (1.2-4.3) | 0.011 | 1.4 (1-1.8) | 0.029 | 1.1 (0.8-1.5) | 0.62 | 1.1 (0.86-1.5) | 0.38 |
| **Donor CMV- (vs. CMV+)** | 2.1 (1.2-3.6) | 0.0092 | 1.7 (1.3-2.2) | < 0.001 | 1 (0.77-1.4) | 0.86 | 0.97 (0.75-1.2) | 0.79 |
| **Complex karyotype (no vs. yes)** | 0.87 (0.41-1.8) | 0.72 | 0.85 (0.59-1.2) | 0.36 | 0.64 (0.42-1) | 0.049 | 1 (0.94-1.2) | 0.4 |
| **Donor: M(M)UD vs. matched-RD** | 2.8 (0.89-9.1) | 0.079 | 1.3 (0.88-2) | 0.18 | 2.3 (1.3-4) | 0.0025 | 1.3 (0.89-1.8) | 0.19 |
| **Age of donor at allo-HCT (continuous)** | 1.1 (1-1.1) | < 0.001 | 1 (0.99-1) | 0.79 | 0.99 (0.98-1) | 0.19 | 0.99 (0.98-1) | 0.23 |
| **MRD positive before allo-HCT (no vs. yes)** | 1 (0.52-1.9) | 0.99 | 1.2 (0.88-1.7) | 0.25 | 0.9 (0.63-1.3) | 0.56 | 0.99 (0.94-1) | 0.55 |
| ***In-vivo* T-cell depletion (no vs. yes)** | 0.099 (0.057-0.17) | < 0.001 | 1.4 (1-2) | 0.042 | 1.6 (1.1-2.4) | 0.017 | 1.2 (0.85-1.7) | 0.31 |
| **Tac vs. CSA** | 2.5 (0.34-18) | 0.37 | 0.45 (0.063-3.2) | 0.43 | 1.1e-07 (0-Inf) | 0.99 | 3.2 (0.8-13) | 0.098 |
| **ELN2017 risk score** | 1.4 (0.91-2.1) | 0.14 | 1.2 (0.94-1.4) | 0.16 | 0.81 (0.65-1) | 0.066 | 1 (0.91-1.2) | 0.68 |
| **IPSSR score** | 1.6 (0.85-2.9) | 0.15 | 0.84 (0.66-1.1) | 0.18 | 0.89 (0.68-1.2) | 0.39 | 0.94 (0.83-1.1) | 0.3 |
| **Days until first stop of IS (continuous)** | 1 (1-1) | 0.35 | 1 (1-1) | 0.031 | 1 (1-1) | 0.89 | 1 (1-1) | 0.059 |
| **IS at day +180 (yes vs. no)** | 0.58 (0.34-1) | 0.048 | 1.4 (1.1-1.8) | 0.021 | 1.2 (0.85-1.6) | 0.33 | 1.7 (1.3-2.2) | < 0.001 |
| **IS at two years (yes vs. no)** | 0.5 (0.27-0.92) | 0.027 | 1.2 (0.85-1.7) | 0.28 | 0.86 (0.59-1.2) | 0.42 | 1.3 (0.92-1.9) | 0.13 |

*HR* hazard ratio, *CI* confidence interval, *FluTreo* fludarabine/treosulfan, *FluTBI* fludarabine/8Gy total body irradiation/fludarabine, *allo-HCT* allogeneic hematopoietic stem cell transplantation, *AML* acute myelogenous leukemia, *MDS* myelodysplastic syndrome, *ECOG* Eastern Cooperative Oncology Group score, *PS* Performance Score, *HCT-CI* hematopoietic cell transplantation-specific comorbidity Index*, CMV* Cytomegalovirus*, MUD* matched unrelated donor*, MMUD* mismatched unrelated donor*, matched-rd* matched related donor*, MRD* measurable residual disease, *Tac* Tacrolimus, *CSA* Ciclosporin A, *ELN2017* European Leukemia Net 2017 classification, *IPSSR* Revised International Prognostic Scoring System for myelodysplastic syndromes risk assessment, *IS* systemic immunosuppression.**Suppl. Table 4. Univariate analysis for post-transplant outcomes for the different conditioning regimens FluTreo vs. FluTBI**

|  | **FluTreo** | **FluTBI** |  |
| --- | --- | --- | --- |
| **Outcomes** | **Probability (95% CI) [%]** | **Probability (95% CI) [%]** | **P-value** |
| **OS** |  |  | 0.061 |
| after 1 yr | 86 (81, 91) | 89 (84, 95) |  |
| after 3 yrs | 71 (65, 79) | 79 (71, 88) |  |
| **RFS** |  |  | 0.240 |
| after 1 yr | 79 (73, 85) | 81 (73, 89) |  |
| after 3 yrs | 63 (56, 71) | 72 (63, 82) |  |
| **Relapse incidence** |  |  | 0.750 |
| after 1 yr | 13 (8.7, 18) | 14 (8.5, 22) |  |
| after 3 yrs | 21 (15, 27) | 21 (13, 30) |  |
| **NRM** |  |  | 0.037 |
| after 1 yr | 8.4 (5.1, 13) | 4.8 (1.8, 10) |  |
| after 3 yrs | 16 (11, 22) | 7 (3.1, 13) |  |
| **aGvHD > grade I** |  |  | 0.294 |
| after 100 ds | 21 (16, 27) | 28 (20, 37) |  |
| after 200 ds | 26 (20, 32) | 36 (26, 45) |  |
| **cGvHD** |  |  | 0.054 |
| after 1 yr | 31 (25, 38) | 21 (14, 29) |  |
| after 3 yrs | 41 (34, 48) | 31 (22, 40) |  |

*FluTreo* fludarabine/treosulfan, *FluTBI* fludarabine/8Gy total body irradiation/fludarabine, *allo-HCT* allogeneic hematopoietic stem cell transplantation, *CI* confidence interval, *OS* overall survival, *RFS* relapse free survival, *NRM* non-relapse mortality, *aGvHD* acute graft versus host disease, *cGvHD* chronic graft versus host disease.

**Table 5. Univariate analysis for post-transplant outcomes for normalization of lymphocyte subpopulations by day +180/ IgG levels at two years after allo-HCT**

|  | **CD3+/CD4+ normalized by day +180** | | | **CD3+/CD8+ normalized by day +180** | | | **CD19+ normalized by day +180** | | | **IgG normalized at two years** | | |
| --- | --- | --- | --- | --- | --- | --- | --- | --- | --- | --- | --- | --- |
|  | yes | no |  | yes | no |  | yes | no |  | yes | no |  |
| **Outcomes** | **Probability (95% CI) [%]** | **Probability (95% CI) [%]** | **P-value** | **Probability (95% CI) [%]** | **Probability (95% CI) [%]** | **P-value** | **Probability (95% CI) [%]** | **Probability (95% CI) [%]** | **P-value** | **Probability (95% CI) [%]** | **Probability (95% CI) [%]** | **P-value** |
| **OS** |  |  | 0.1 |  |  | 0.013 |  |  | <0.001 |  |  | <0.001 |
| after 1 year | 89 (81, 98) | 91 (88, 95) |  | 93 (90, 96) | 79 (68, 92) |  | 96 (93, 99) | 81 (73, 89) |  | 96 (93, 98) | 69 (57, 83) |  |
| after 3 years | 64 (51, 82) | 79 (73, 85) |  | 78 (73, 85) | 64 (50, 82) |  | 82 (76, 89) | 65 (55, 76) |  | 83 (78, 89) | 51 (38, 70) |  |
| **RFS** |  |  | 0.09 |  |  | 0.2 |  |  | 0.002 |  |  | <0.001 |
| after 1 year | 87 (79, 97) | 84 (79, 89) |  | 86 (81, 90) | 77 (65, 91) |  | 89 (85, 94) | 75 (66, 84) |  | 89 (85, 93) | 64 (52, 78) |  |
| after 3 years | 57 (43, 75) | 74 (68, 81) |  | 73 (67, 79) | 61 (46, 79) |  | 77 (70, 84) | 59 (49, 71) |  | 76 (70, 82) | 50 (38, 67) |  |
| **Relapse incidence** |  |  | >0.9 |  |  | 0.2 |  |  | 0.075 |  |  | 0.4 |
| after 1 year | 7.3 (2.3, 16) | 12 (8.4, 17) |  | 12 (8, 16) | 9.2 (2.9, 20) |  | 9 (5.4, 14) | 16 (9.3, 24) |  | 10 (6.9, 15) | 17 (8.4, 29) |  |
| after 3 years | 17 (7.6, 30) | 18 (13, 23) |  | 18 (13, 24) | 13 (4.4, 27) |  | 15 (9.6, 20) | 24 (15, 33) |  | 18 (13, 23) | 23 (12, 36) |  |
| **NRM** |  |  | 0.007 |  |  | 0.002 |  |  | 0.031 |  |  | <0.001 |
| after 1 year | 5.5 (1.4, 14) | 4.1 (2, 7.4) |  | 2.6 (1.1, 5.3) | 14 (5.6, 26) |  | 1.7 (0.5, 4.5) | 9.5 (4.7, 17) |  | 0.9 (0.2, 2.9) | 19 (9.8, 31) |  |
| after 3 years | 26 (13, 41) | 8.3 (4.9, 13) |  | 9.1 (5.4, 14) | 26 (13, 41) |  | 8.5 (4.5, 14) | 18 (10, 27) |  | 6.6 (3.5, 11) | 27 (15, 40) |  |
| **CMV react.** |  |  | 0.8 |  |  | 0.9 |  |  | 0.3 |  |  | 0.019 |
| after 1 year | 13 (5.5, 23) | 19 (14, 24) |  | 17 (12, 22) | 20 (9.2, 34) |  | 14 (9.8, 20) | 23 (15, 32) |  | 15 (11, 20) | 31 (18, 45) |  |
| after 3 years | 20 (10, 33) | 20 (15, 26) |  | 20 (15, 26) | 20 (9.2, 34) |  | 18 (12, 24) | 25 (16, 34) |  | 17 (13, 23) | 36 (21, 52) |  |
| **CMV/ EBV/ HSV react.** |  |  | 0.8 |  |  | 0.8 |  |  | 0.3 |  |  | 0.012 |
| after 1 year | 13 (5.6, 23) | 19 (14, 24) |  | 17 (13, 22) | 20 (9.3, 34) |  | 15 (9.9, 20) | 24 (15, 33) |  | 18 (14, 23) | 35 (21, 48) |  |
| after 3 years | 21 (10, 33) | 20 (15, 26) |  | 20 (15, 26) | 20 (9.3, 34) |  | 18 (12, 24) | 25 (17, 35) |  | 29 (23, 36) | 49 (30, 65) |  |
| **aGvHD > grade I** |  |  | 0.14 |  |  | 0.006 |  |  | 0.2 |  |  | <0.001 |
| after 100 days | 22 (12, 34) | 24 (18, 29) |  | 21 (16, 27) | 33 (19, 47) |  | 22 (16, 28) | 26 (18, 35) |  | 19 (15, 25) | 42 (28, 54) |  |
| after 200 days | 22 (12, 34) | 31 (25, 37) |  | 26 (21, 32) | 45 (30, 60) |  | 27 (21, 34) | 33 (23, 42) |  | 25 (20, 31) | 51 (37, 64) |  |
| **cGvHD** |  |  | <0.001 |  |  | 0.6 |  |  | 0.007 |  |  | 0.4 |
| after 1 year | 47 (32, 60) | 23 (18, 29) |  | 26 (21, 32) | 34 (19, 49) |  | 24 (18, 30) | 35 (25, 45) |  | 28 (22, 34) | 37 (24, 51) |  |
| after 3 years | 72 (55, 84) | 37 (30, 45) |  | 44 (37, 51) | 41 (24, 56) |  | 38 (31, 46) | 56 (43, 68) |  | 42 (35, 49) | 41 (26, 56) |  |

*CI* confidence interval, *allo-HCT* allogeneic hematopoietic stem cell transplantation, *OS* overall survival, *RFS* relapse free survival, *NRM* non-relapse mortality, *CMV* cytomegalovirus, *react.* reactivation, *EBV* Epstein-Barr virus, *HSV* herpes simplex virus, *aGvHD* acute graft versus host disease, *cGvHD* chronic graft versus host disease.

**Suppl. Table 6. Numeric reasons for NRM in patients with or without normalization of lymphocyte subpopulations by day +180/ IgG levels by two years after allo-HCT**

|  | **CD3+/CD4+ normalized until d+180** | | **CD3+/CD8+ normalized until d+180** | | **CD19+ normalized until d+180** | | **IgG normalized until d+720** | |
| --- | --- | --- | --- | --- | --- | --- | --- | --- |
|  | yes | no | yes | no | yes | no | yes | no |
|  |  |  |  |  |  |  |  |  |
| **GvHD** | 1 | 3 | 2 | 2 | 2 | 2 | 3 | 2 |
| **Infection** | 5 | 1 | 5 | 1 | 4 | 5 | 2 | 3 |
| **Infection + GvHD** | 2 | 7 | 6 | 4 | 1 | 5 | 5 | 5 |
| **NOMI** | 1 | 0 | 0 | 1 | 0 | 1 | 0 | 1 |
| **Cardiogenic shock** | 1 | 0 | 0 | 1 | 1 | 0 | 1 | 0 |
| **Pulmonary embolism** | 1 | 0 | 1 | 0 | 1 | 0 | 0 | 0 |
| **Second malignancy** | 1 | 0 | 1 | 0 | 0 | 1 | 0 | 0 |
| **unknown** | 3 | 0 | 2 | 1 | 2 | 1 | 2 | 2 |

*Allo-HCT* allogeneic hematopoietic stem cell transplantation, *GvHD* graft-versus-host disease*, NOMI* non-occlusive mesenteric ischemia.

**Suppl. Table 7. Acute and long-term toxicities after allo-HCT by conditioning regimen**

|  | **FluTreo** | **FluTBI** | **P-value** |
| --- | --- | --- | --- |
| Acute pulmonary toxicity | 0% | 4.3% | 0.07 |
| Acute hepatic toxicity | 3.8% | 3.8% | 1 |
| Acute dermal toxicity | 4.3% | 1.9% | 0.44 |
| Oral mucositis (any grade) | 42.5% | 64.4% | <0.001 |
| Oral mucositis (CTCAE grade ≥ 2) | 28% | 57.7% | <0.001 |
| Oral mucositis (CTCAE grade ≥ 3) | 13.5% | 31.7% | <0.001 |
| Long-term pulmonary toxicity | 3.9% | 1.9% | 0.25 |
| Long-term hepatic toxicity | 1.4% | 1% | 1 |
| Long-term dermal toxicity | 2.9% | 5.8% | 0.35 |
| Other long-term toxicity | 13.5% | 17.3% | 0.47 |
| CMV reactivation | 17.9% | 23.1% | 0.48 |
| Reactivation of CMV/ EBV/ HSV | 13% | 19.2% | 0.31 |
| Reactivation of any virus | 27.5% | 36.5% | 0.22 |
| Secondary malignancies | 2.95% | 7.7% | 0.1 |

*FluTreo* fludarabine/treosulfan, *FluTBI* fludarabine/8Gy total body irradiation/fludarabine, *allo-HCT* allogeneic hematopoietic stem cell transplantation, *CTCAE* common toxicity criteria for adverse events, *CMV* cytomegalovirus*, EBV* Epstein-Barr virus, *HSV* herpes simplex virus.

**References**

1. Berning P, Kolloch L, Reicherts C, Call S, Marx J, Floeth M, et al. Comparable outcomes for TBI-based versus treosulfan based conditioning prior to allogeneic hematopoietic stem cell transplantation in AML and MDS patients. Bone Marrow Transplant. 2024;59(8):1097-106.

2. Dohner H, Estey E, Grimwade D, Amadori S, Appelbaum FR, Buchner T, et al. Diagnosis and management of AML in adults: 2017 ELN recommendations from an international expert panel. Blood. 2017;129(4):424-47.

3. Greenberg PL, Tuechler H, Schanz J, Sanz G, Garcia-Manero G, Sole F, et al. Revised international prognostic scoring system for myelodysplastic syndromes. Blood. 2012;120(12):2454-65.
